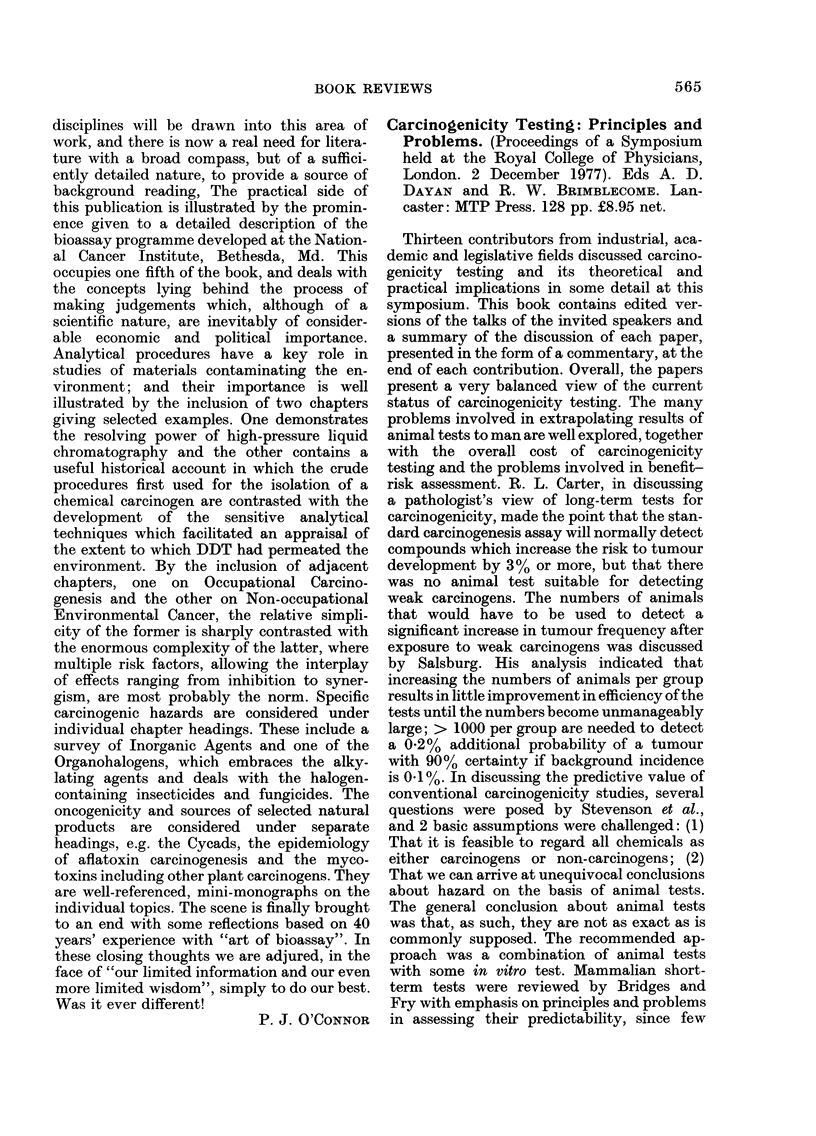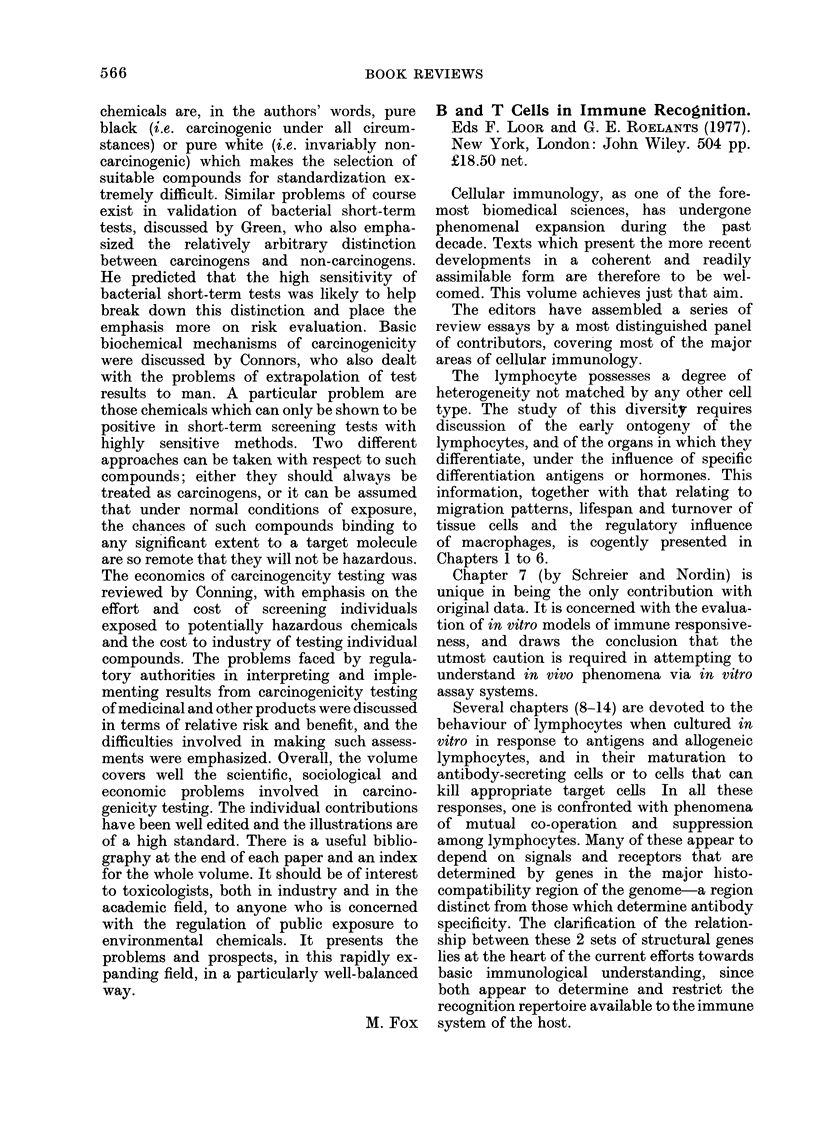# Carcinogenicity Testing: Principles and Problems

**Published:** 1978-10

**Authors:** M. Fox


**Carcinogenicity Testing: Principles and Problems.** (Proceedings of a Symposium held at the Royal College of Physicians, London. 2 December 1977). Eds A. D. Dayan and R. W. Brimblecome. Lancaster: MTP Press. 128 pp. £8.95 net.

Thirteen contributors from industrial, academic and legislative fields discussed carcinogenicity testing and its theoretical and practical implications in some detail at this symposium. This book contains edited versions of the talks of the invited speakers and a summary of the discussion of each paper, presented in the form of a commentary, at the end of each contribution. Overall, the papers present a very balanced view of the current status of carcinogenicity testing. The many problems involved in extrapolating results of animal tests to man are well explored, together with the overall cost of carcinogenicity testing and the problems involved in benefit–risk assessment. R. L. Carter, in discussing a pathologist's view of long-term tests for carcinogenicity, made the point that the standard carcinogenesis assay will normally detect compounds which increase the risk to tumour development by 3% or more, but that there was no animal test suitable for detecting weak carcinogens. The numbers of animals that would have to be used to detect a significant increase in tumour frequency after exposure to weak carcinogens was discussed by Salsburg. His analysis indicated that increasing the numbers of animals per group results in little improvement in efficiency of the tests until the numbers become unmanageably large; $>$ 1000 per group are needed to detect a 0·2% additional probability of a tumour with 90% certainty if background incidence is 0·1%. In discussing the predictive value of conventional carcinogenicity studies, several questions were posed by Stevenson *et al.*, and 2 basic assumptions were challenged: (1) That it is feasible to regard all chemicals as either carcinogens or non-carcinogens; (2) That we can arrive at unequivocal conclusions about hazard on the basis of animal tests. The general conclusion about animal tests was that, as such, they are not as exact as is commonly supposed. The recommended approach was a combination of animal tests with some *in vitro* test. Mammalian short-term tests were reviewed by Bridges and Fry with emphasis on principles and problems in assessing their predictability, since few chemicals are, in the authors' words, pure black (*i.e.* carcinogenic under all circumstances) or pure white (*i.e.* invariably non-carcinogenic) which makes the selection of suitable compounds for standardization extremely difficult. Similar problems of course exist in validation of bacterial short-term tests, discussed by Green, who also emphasized the relatively arbitrary distinction between carcinogens and non-carcinogens. He predicted that the high sensitivity of bacterial short-term tests was likely to help break down this distinction and place the emphasis more on risk evaluation. Basic biochemical mechanisms of carcinogenicity were discussed by Connors, who also dealt with the problems of extrapolation of test results to man. A particular problem are those chemicals which can only be shown to be positive in short-term screening tests with highly sensitive methods. Two different approaches can be taken with respect to such compounds; either they should always be treated as carcinogens, or it can be assumed that under normal conditions of exposure, the chances of such compounds binding to any significant extent to a target molecule are so remote that they will not be hazardous. The economics of carcinogencity testing was reviewed by Conning, with emphasis on the effort and cost of screening individuals exposed to potentially hazardous chemicals and the cost to industry of testing individual compounds. The problems faced by regulatory authorities in interpreting and implementing results from carcinogenicity testing of medicinal and other products were discussed in terms of relative risk and benefit, and the difficulties involved in making such assessments were emphasized. Overall, the volume covers well the scientific, sociological and economic problems involved in carcinogenicity testing. The individual contributions have been well edited and the illustrations are of a high standard. There is a useful bibliography at the end of each paper and an index for the whole volume. It should be of interest to toxicologists, both in industry and in the academic field, to anyone who is concerned with the regulation of public exposure to environmental chemicals. It presents the problems and prospects, in this rapidly expanding field, in a particularly well-balanced way.

M. FOX